# Supplementary material for: Identification of the FSH-RH as the other gonadotropin-releasing hormone
Source: Nat Commun. 2024 Jun 27;15:5342. doi: 10.1038/s41467-024-49564-8 (PMC11211334; doi:10.1038/s41467-024-49564-8)
Supplement: Supplementary file 1 — Supplementary Information [file 41467_2024_49564_MOESM1_ESM.pdf]

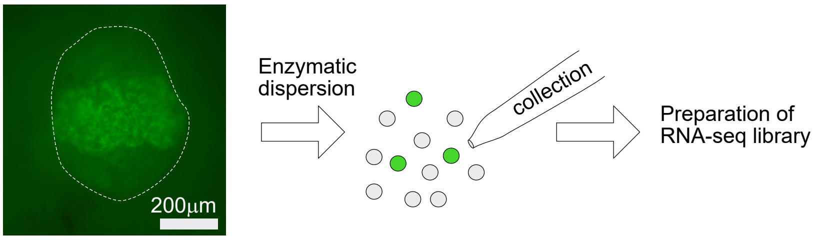

Supplementary Fig. 1. Schematic of FSH-specific RNA-seq by using FSH-GFP transgenic medaka.
Left fluorescent image is the pituitary of the FSH-GFP transgenic medaka. Sub-region where FSH cells are localized show GFP fluorescence. Dotted line indicates the edge of the whole pituitary. The pituitary of the FSH-GFP transgenic medaka was excised and subjected to enzymatic dispersion. Then, the GFP positive FSH cells were collected and RNA-seq was performed.

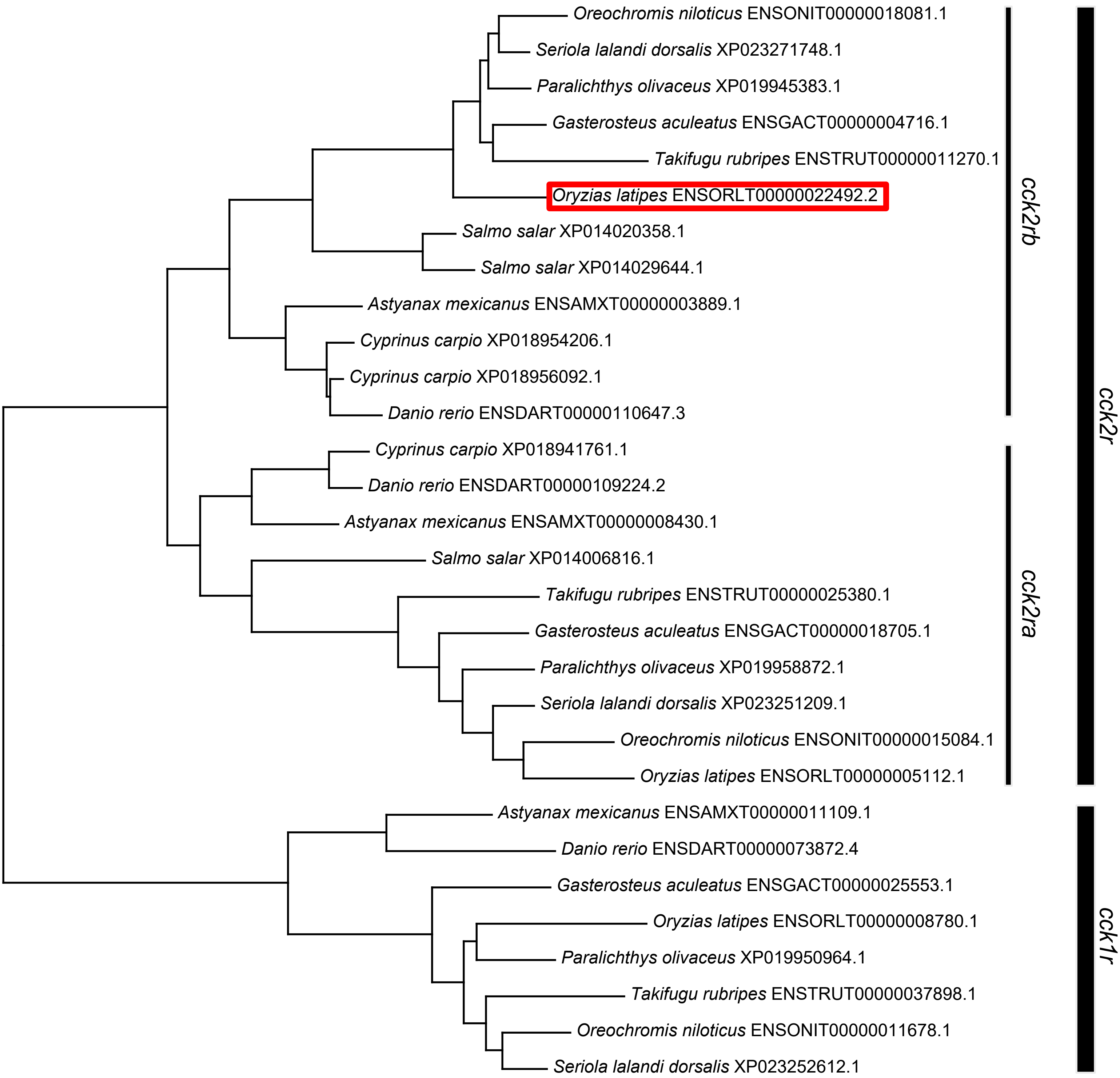

Supplementary Fig. 2. Phylogenetic tree of cholecystokinin (cck1 and cck2) receptors.

Maximum likelihood tree of the CCK receptors based on a part of deduced amino acid sequence of each gene. Species name and the accession number for each species are indicated in the tree.

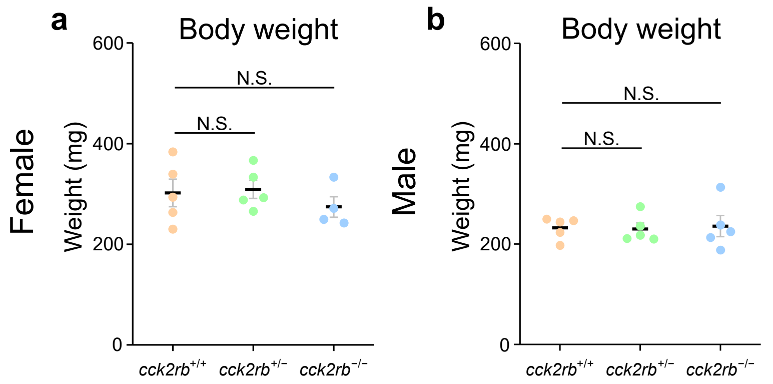

Supplementary Fig. 3. The body weight does not change among *cck2rb*$^{+/+}$, *cck2rb*$^{+/-}$, and *cck2rb*$^{-/-}$.

a,b Body weight of *cck2rb*$^{+/+}$, *cck2rb*$^{+/-}$, and *cck2rb*$^{-/-}$ female (a) and male (b) medaka (n = 5 fish). No significant change of body weight was observed among them. The data are mean ± SEM. N.S., not significant, two-sided Dunnett's test (**a**,**b**). Source data are provided as a Source Data file.

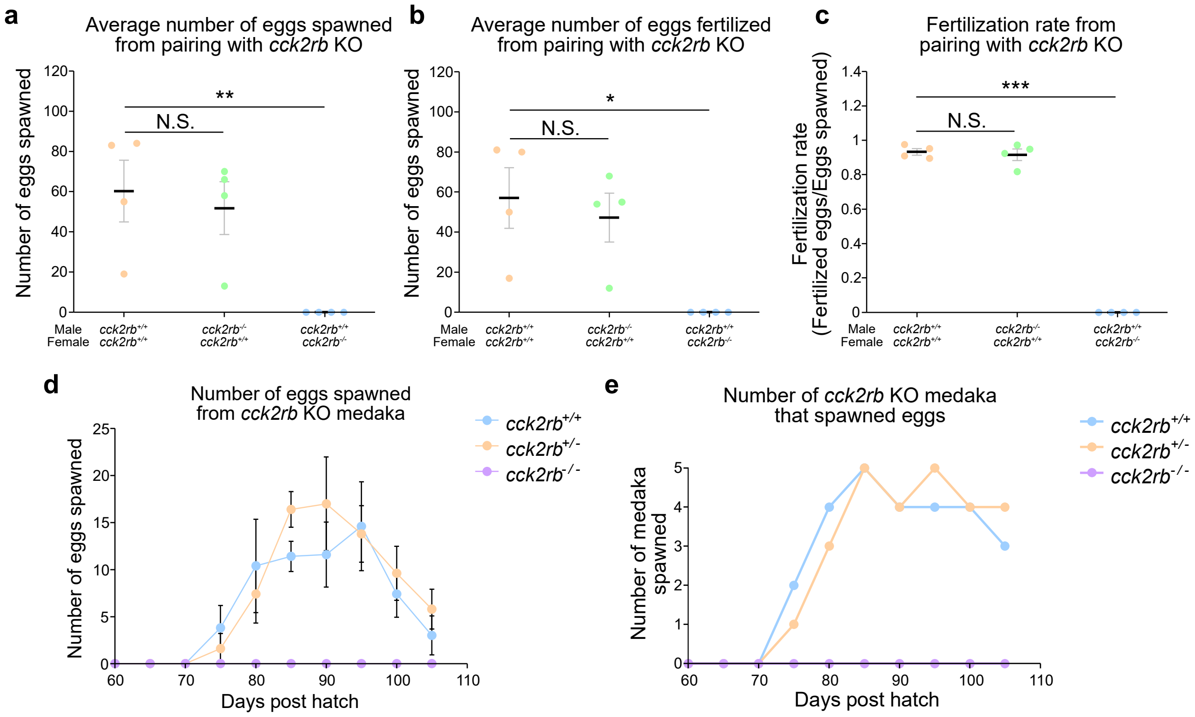

Supplementary Fig. 4. Eggs spawned from *cck2rb* medaka of each genotype.
a,b The number of eggs spawned (a) and fertilized eggs (b) from wild type or *cck2rb*$^{-/-}$ female medaka paired with wild type or *cck2rb*$^{-/-}$ male medaka (n = 4 pairs; **, p = 0.0096; *, p = 0.0011). The data are mean ± SEM. **c** The fertilization rate of each paired groups determined by the number of eggs spawned over the number of fertilized eggs (n = 4 pairs; ***, p = 2.1e-6). The data are mean ± SEM. **d** The number of eggs spawned from female of each genotype paired with wild type male (n = 5 pairs). The data are mean ± SEM. **e** The number of females that spawned eggs in each genotype when paired with a wild type male. No eggs were spawned from *cck2rb*$^{-/-}$ while *cck2rb*$^{+/+}$ and *cck2rb*$^{+/-}$ spawned. *p<0.05, **p<0.01, ***p<0.001, N.S., not significant, two-sided Dunnett's test (**a-c**). Source data are provided as a Source Data file.

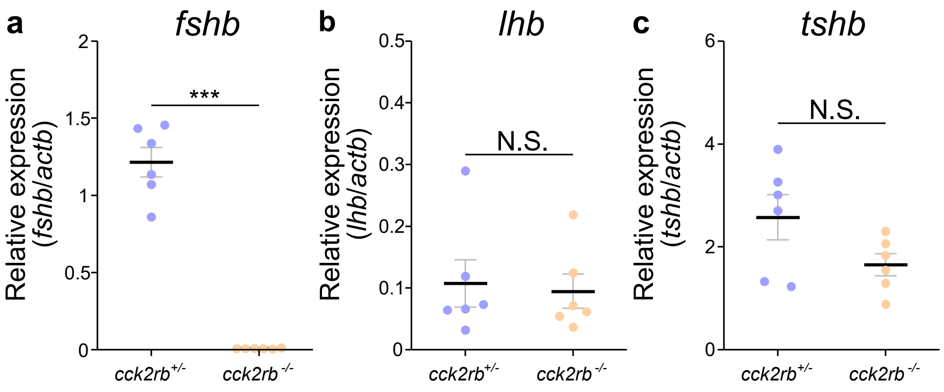

Supplementary Fig. 5. *lhb* expression in *cck2rb*$^{-/-}$ shows similar level as that in *cck2rb*$^{+/-}$ in ovariectomized fish, suggesting that the reduction of *lhb* in *cck2rb* KO should be a secondary effect.

The effect of *cck2rb* knockout in ovariectomized (OVX) females (n = 6 fish). **a** *fshb* mRNA expression of the pituitary of *cck2rb*$^{-/-}$ by quantitative real-time polymerase chain reaction (qRT-PCR). **b** *lhb* mRNA expression of *cck2rb*$^{+/-}$ and *cck2rb*$^{-/-}$. **c** *tshb* expression of the pituitary extracted from both genotype. The data are mean ± SEM. ***, p = 5.5e-5; N.S., not significant; two-sided Student's t-test (**a-c**). Source data are provided as a Source Data file.

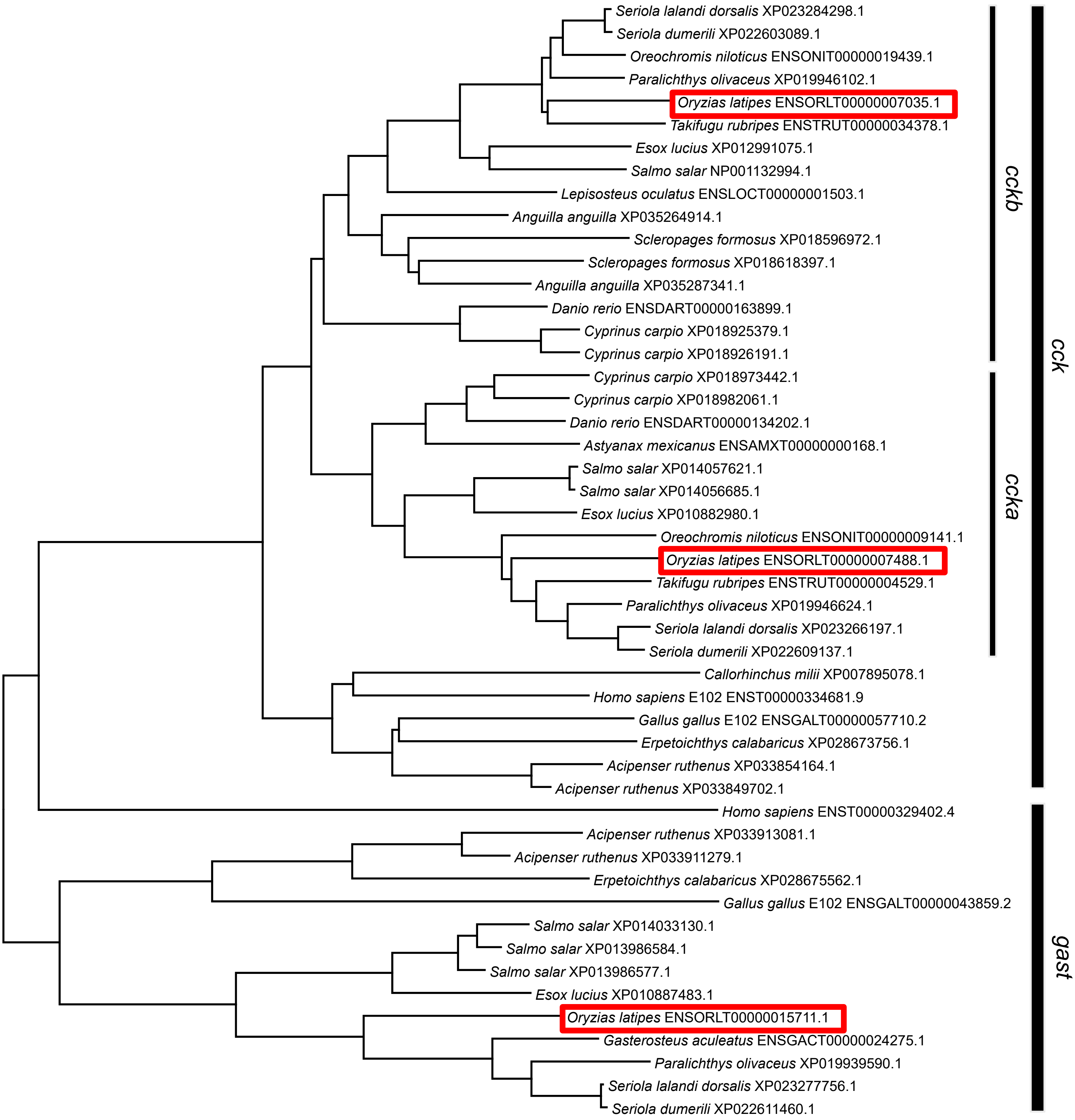

Supplementary Fig. 6. Phylogenetic tree of medaka cholecystokinin (*ccka* and *cckb*) and gastrin (*gast*).

Maximum likelihood tree of the *ccka*, *cckb*, and *gast* based on deduced amino acid sequence. Species name and the accession number for each species are indicated in the tree.

```
                          *         20         *
ChickCck   : MYGG-ICICVLLAALSVSSLGQQPAGSHDGSP------ :  31
GarCck     : MNSG-ICVCVLLAVLSTSCLGRPASGSEDEG------- :  30
ZebraCcka  : MNAG-LCVCALLAALSTSSCLSLPVHSEDG-------- :  29
MedakaCcka : MNVG-IYVCVILAALFTGSLTLPSKSMFQR-------- :  29
ZebraCckb  : MNSG-VCVCVILAALSVS-----VSCASR--------- :  23
MedakaCckb : MTAG-LGVCVVLAVLCTSCLGLPVSSAPSDEGQHSLSA :  37
ChickGast  : MKTK-VFLGLILSAAVTACLCRPAAKAPGGS------- :  30
GarGast    : MPGSKVCLCALLVTVLAAVCLAVPLPETLGD------- :  31
ZebraGast  : MMAKFIVLT--IVAMLVAACAASPLSK----------- :  25
MedakaGast : MSGKTALLFALLVVLLVSSSASPAKAE--GG------- :  29

             40         *         60         *
ChickCck   : ---VAAELQQSLTEPHRHSR---APSSAGPLKPAPRL- :  62
GarCck     : ---TPAQLGQSLSAPRRTAR---SAPPHGQPQPFQRA- :  61
ZebraCcka  : ---VQSNVG-SATGHTRHTR---AAPPAGQINLLTKP- :  59
MedakaCcka : ---TERKALVTESLPVPLTN---HTRQARSAPAPPSGQ :  61
ZebraCckb  : ------PVSDERSLSARRLA---RSASLTLQQPLPPAG :  52
MedakaCckb : PSEVALPEADTKSLDGAHVR---HSRSTTQLKDLPGA- :  71
ChickGast  : -----------------------HRPNSSLAR------ :  39
GarGast    : ---AGAALAHRDALRERARALRERTPSGAPRDPAQGAR :  66
ZebraGast  : ---VKPVNTKRSA----------ISPESSPE--AHETP :  48
MedakaGast : ---TG-ALAHKGA-----------EPTGRMR------A :  46

               80         *        100         *
ChickCck   : DGSFEQRATIGALLAKYLQQAWKGSTGRFSVLGNRVQS : 100
GarCck     : EEAAEQRASLGELLARLLNR--KGSFRRNSTANSKASG :  97
ZebraCcka  : EDDEEPRSSLTELLARIIST--KGSYRRSPAANSRT-- :  93
MedakaCcka : LQSESAQNSLSQLLARLLSRK-SSSLQTRSSLTSR--A :  96
ZebraCckb  : DIQPDTRANLSQLLAKLISSK-KGSVRRNSSMNSR--A :  87
MedakaCckb : EEDGDSRANLSELLARLISTR-KGSVRRNSSANNRGGV : 108
ChickGast  : --RDWPEPPSQEQQQRFISRFLPHVFAELSDRKGFVQG :  75
GarGast    : VARQDRLGSLTEEQRDLVSRYVLQALTELAHREGCS-- : 102
ZebraGast  : LARVRRSVGLSEDQRELMSRQLLQALSEIIQREDC--- :  83
MedakaGast : ATRERRRAHLTEDEREMMTKQIVQALSEVMNSD-C--- :  80

                120         *        140
ChickCck   : IDPTHRINDRDYMGWMDFGRRSAEEYEYSS : 130
GarCck     : LSANHRIKDRDYLGWMDFGRRSAEEYEYSS : 127
ZebraCcka  : MGASHRIKDRDYLGWMDFGRRSAEEYEYSS : 123
MedakaCcka : AAPSHRIKDRDYLGWMDFGRRSVEEYEYSP : 126
ZebraCckb  : NSVNHRIKDRDYVGWMDFGRRSAEEYEYSS : 117
MedakaCckb : LRANHRIADRDYLGWMDFGRRSAEEYEYSS : 138
ChickGast  : NGAVEALHDHFYPDWMDFGRRSTEDAADAA : 105
GarGast    : DDHPINISDRDYHGWMDFGRRSAEELDS-- : 130
ZebraGast  : ------LS--DYQGWVDFGRRSSN------ :  99
MedakaGast : ------MSDRDYRGWLDFGRRDAE------ :  98
```

Supplementary Fig. 7. Alignment of amino acid sequence of vertebrate CCK and gastrin.

Alignment of medaka (MedakaCcka, ENSORLT00000007488.1; MedakaCckb, ENSORLT00000007035.1; gast, ENSORLT00000015711.1) compared with zebrafish (ZebraCcka, ENSDART00000134202.1; ZebraCckb, ENSDART00000163899.1; ZebraGast, XP_021335429.1), gar (GarCck, ENSLOCT00000001503.1; GarGast, XP_006638439.1) and chicken(ChickCck, ENSGALT00000057710.2; ChickGast, ENSGALT00000043859.2). The red horizontal line indicates the C-terminal CCK octapeptide (CCK-8). Black shading represents identical residues while gray shading represents similar residues.

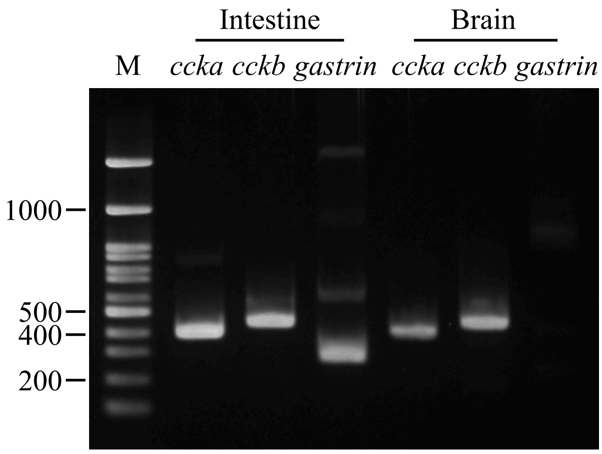

Supplementary Fig. 8. ***ccka*** **and** ***cckb*** **but not** ***gast*** **is expressed in the brain.**
Reverse transcription polymerase chain reaction (RT-PCR) analysis of the medaka brain and intestine. cDNA of brain and intestine were examined for PCR using *ccka*- (381-bp), *cckb*- (437-bp), and *gast*- (295-bp) specific primers and resolved in 2% agarose gel electrophoresis. *ccka* and *cckb* were amplified from both brain and intestine cDNA, whereas *gast* was amplified only from the intestine cDNA. Source data are provided as a Source Data file.

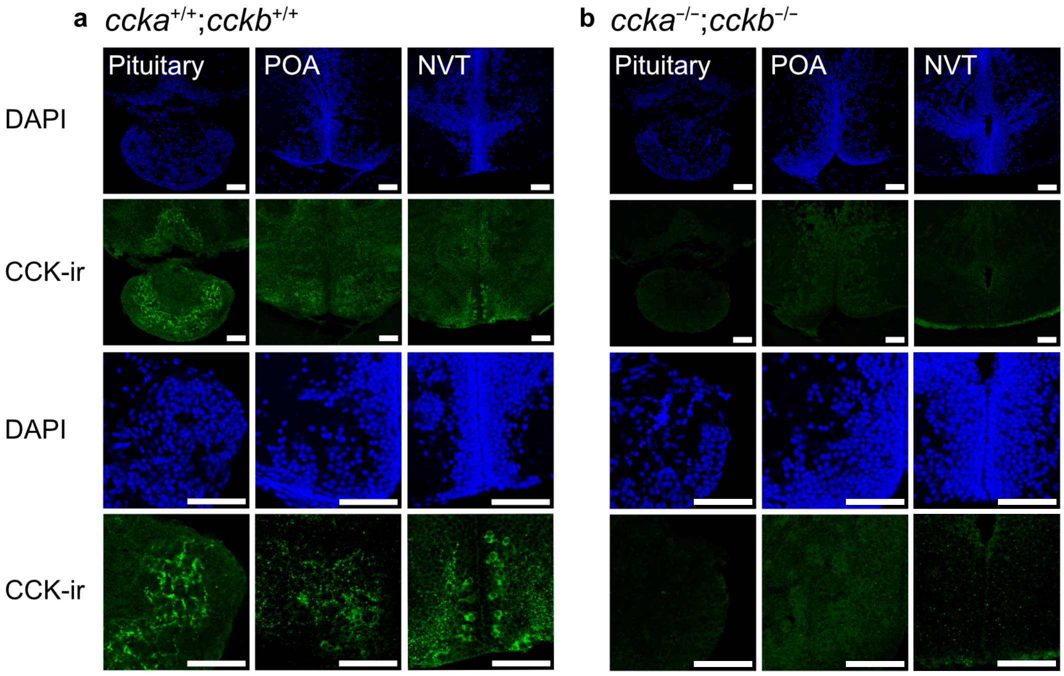

Supplementary Fig. 9. *ccka* and *cckb* knockout fish do not have CCK-immunoreactive cell bodies and fibers.

a Immunohistochemistry of *ccka* and *cckb* double knockout medaka, using CCK antibody. Cell bodies and fibers were observed in the pituitary, preoptic area (POA), and nucleus ventralis tuberis (NVT) of *ccka*$^{+/+}$;*cckb*$^{+/+}$ medaka. **b** No cell bodies or fibers were observed in *ccka*$^{-/-}$; *cckb*$^{-/-}$ medaka.The bottom half of the figures are in higher magnification. Scale bars, 50 µm.

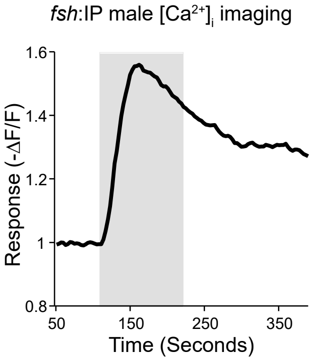

Supplementary Fig. 10. Increased $[Ca^{2+}]_i$ in FSH cells in male in response to CCK-8s peptide.
FSH cells in male was applied with 1000 nM CCK-8s peptide. The shaded section indicates the time and duration of the perfusion of CCK-8s. During the application of peptide, FSH cells showed drastic increase of $[Ca^{2+}]_i$ which is similar to the response in females. Source data are provided as a Source Data file.

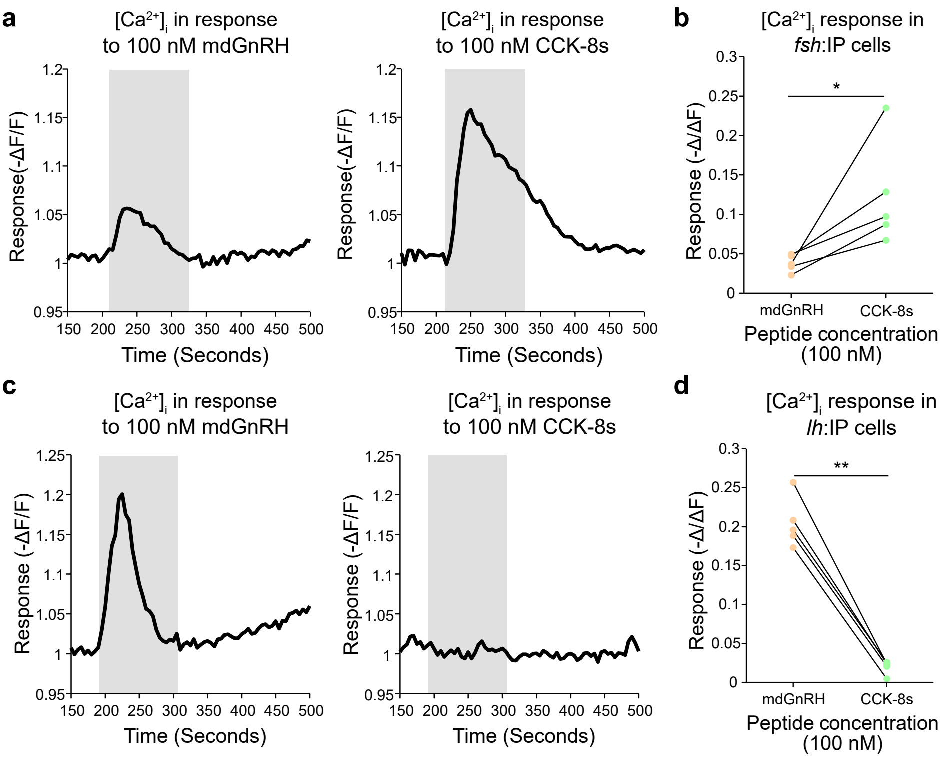

Supplementary Fig. 11. $[Ca^{2+}]_i$ of FSH cells when perfused with 100 nM mdGnRH or 100 nM CCK-8s peptide.

a Ca^{2+} imaging of FSH cells applied with 100 nM mdGnRH or 100 nM CCK-8s. The shaded section indicates the time and duration of the perfusion of the peptide.

b The response of FSH cells to CCK-8s peptide is significantly greater than that to mdGnRH at 100 nM (n = 5 fish; *, $p = 0.022$). **c** Ca^{2+} imaging of LH cells applied with 100 nM mdGnRH or 100 nM CCK-8s. The shaded section indicates the time and duration of the perfusion of the peptide. **d** The response of LH cells to mdGnRH is significantly greater that that to CCK-8s at 100 nM (n = 5 fish; **, $p = 0.0012$). *$p<0.05$, **$p<0.01$, two-sided Student's t-test (**b**, **d**). Source data are provided as a Source Data file.

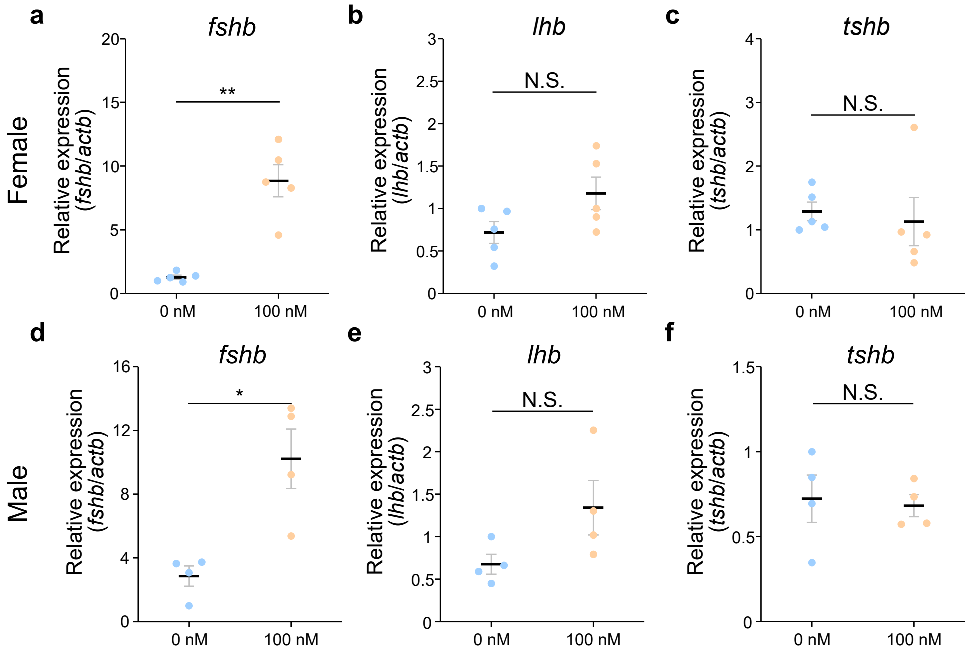

Supplementary Fig. 12. CCK-8s exclusively increases the *fshb* expression. Since there might be a increasing trend in *lhb* expression after incubation with CCK-8s, similar experiments was reexamined for both female and male. **a-f** qRT-PCR of the pituitaries after incubating in CCK-8s for 48 hours (a and d, *fshb*; b and e, *lhb*; c and f, *tshb*; n = 5, 4 fish). Only *fshb* expression was significantly different between the pituitary incubated with or without 100 nM CCK-8s. The data are mean ± SEM. $**p = 0.0057$, $*p = 0.014$, N.S., not significant, two-sided Student's t-test (**a-f**). Source data are provided as a Source Data file.

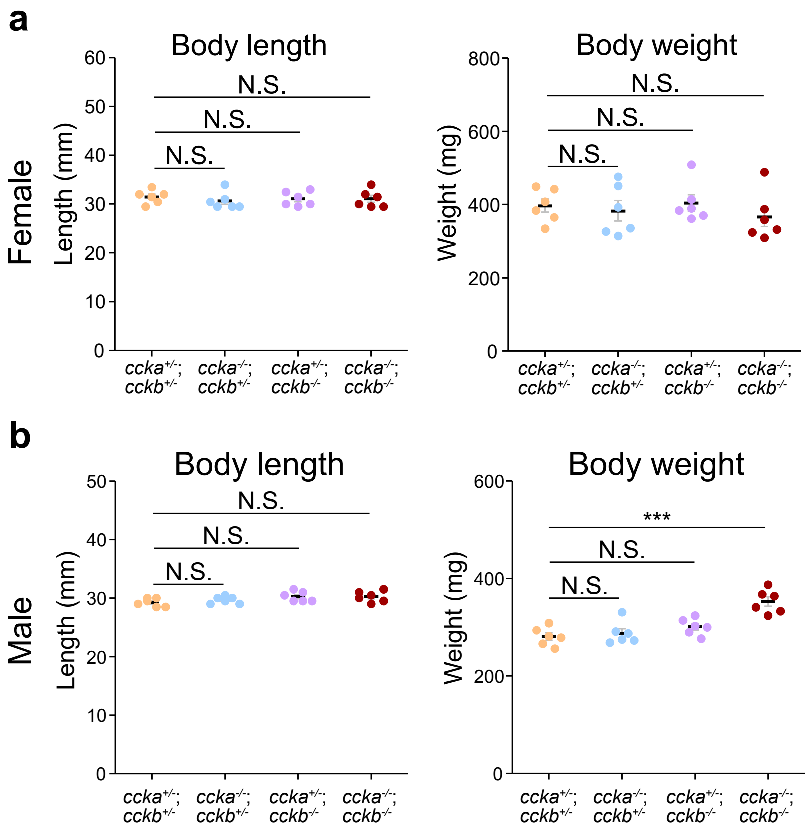

Supplementary Fig. 13. Body length and weight are mostly same among *ccka* and *cckb* double KO.

a The body length and weight of *ccka*$^{+/-}$;*cckb*$^{+/-}$, *ccka*$^{-/-}$;*cckb*$^{+/-}$, *ccka*$^{+/-}$;*cckb*$^{-/-}$, and *ccka*$^{-/-}$;*cckb*$^{-/-}$ female medaka (n = 6 fish). **b** The body length and weight of male *ccka*/*cckb* double KO (n = 6 fish). The data are mean ± SEM. ***, p =2.41e-5; N.S., not significant; two-sided Dunnett's test (**a**,**b**). Source data are provided as a Source Data file.

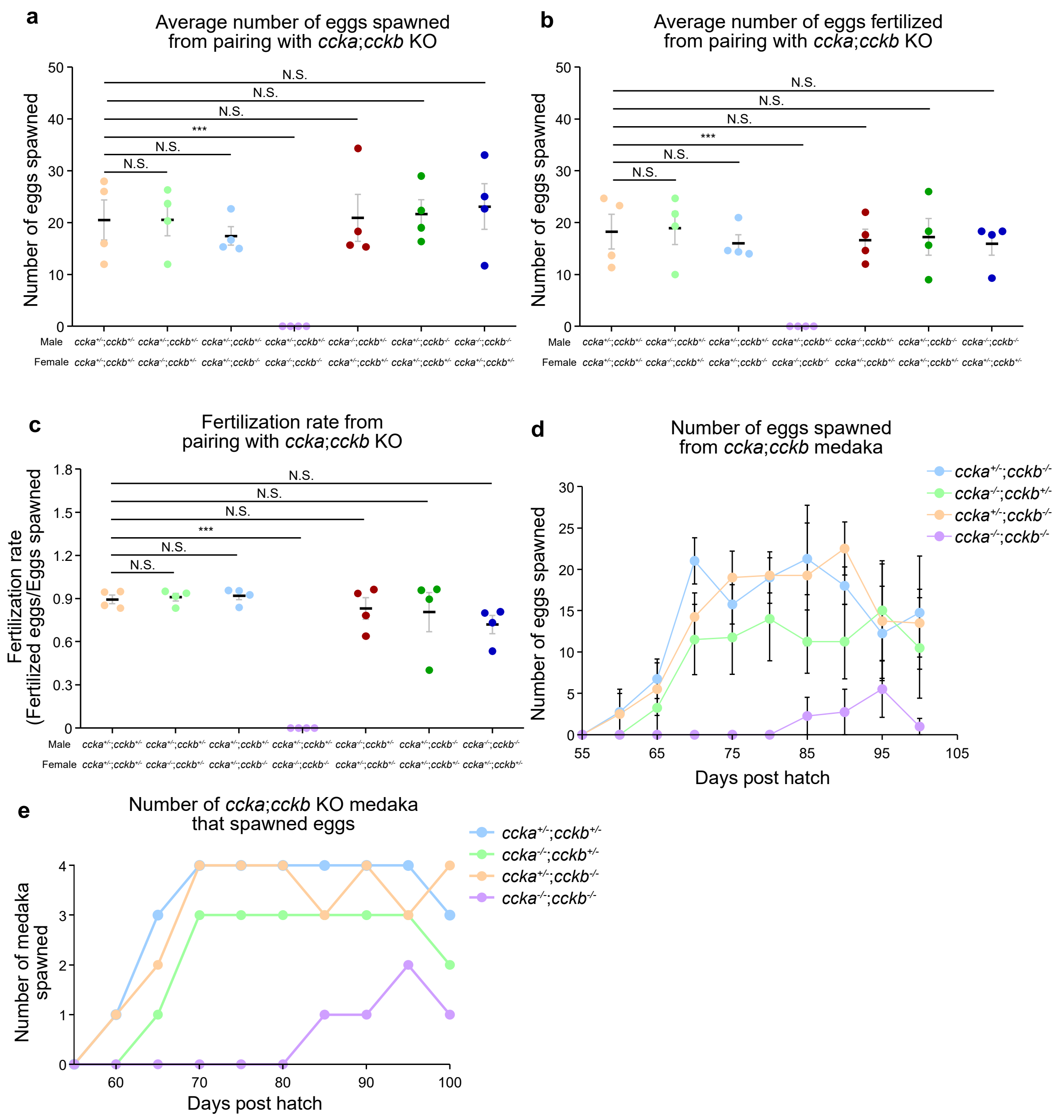

Supplementary Fig. 14. Eggs spawned from *ccka*;*cckb* medaka of each genotype
a,b The number of eggs spawned (a) and fertilized eggs (b) from wild type or *ccka*;*cckb* KO female medaka paired with wild type or *ccka*;*cckb* KO male medaka (n = 4 pairs; ***, p = 0.0012, 3.0e-4). The data are mean ± SEM. **c** The fertilization rate of each paired groups determined by the number of eggs spawned over the number of fertilized eggs (n = 4 pairs; ***, p = 1.4e-6). The data are mean ± SEM. **d** The number of eggs spawned from female of each genotype paired with wild type male (n = 4 pairs). The data are mean ± SEM. **e** The number of females that spawned eggs in each genotype. *ccka*$^{-/-}$;*cckb*$^{-/-}$ medaka began to lay eggs about one month after the other genotypes. ***p<0.001, N.S., not significant, two-sided Dunnett's test (**a-c**). Source data are provided as a Source Data file.

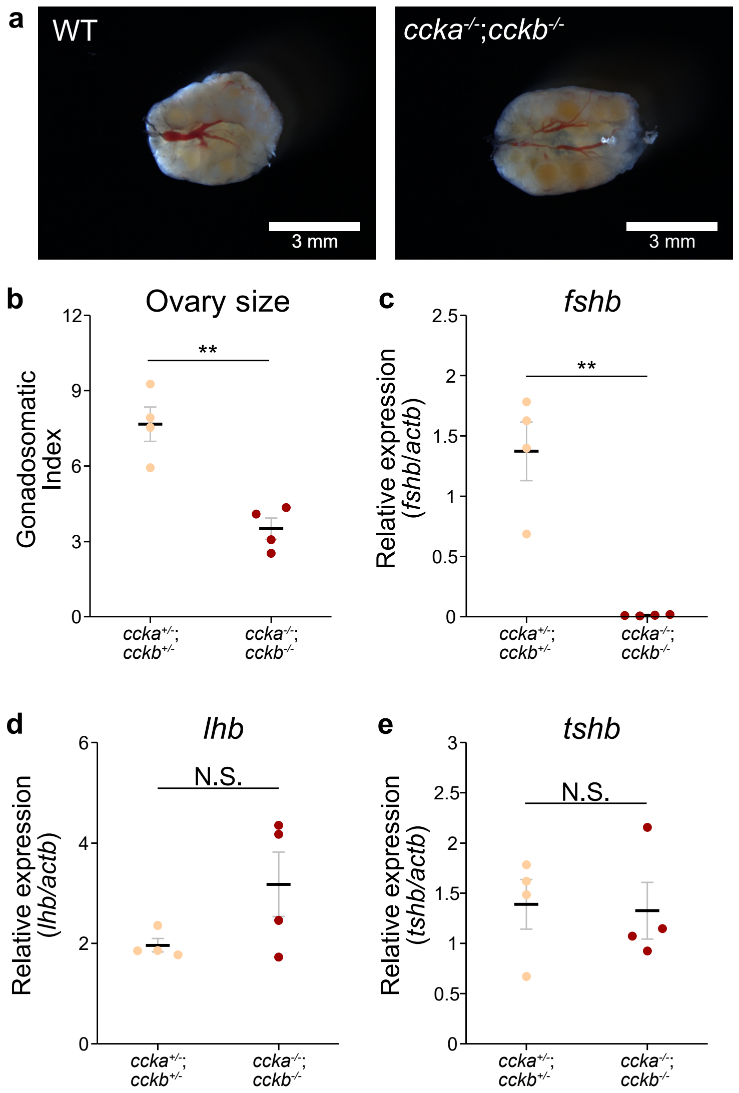

Supplementary Fig. 15. ***ccka/cckb*** **double KO females show delayed spawning with their drastically reduced FSH expression**

a *ccka*$^{-/-}$;*cckb*$^{-/-}$ showed functional ovary after a delay of ~1 month. **b** The gonadosomatic index (GSI) of ovary size normalized by body weight (n = 4 fish; **, p = 0.0021). **c,d** Quantitative real-time polymerase chain reaction (qRT-PCR) of *fshb* expression (c) and *lhb* expression (d) in *ccka*$^{+/-}$;*cckb*$^{+/-}$ and *ccka*$^{-/-}$;*cckb*$^{-/-}$. **e** *tshb* expression of both genotype (n = 4 fish; **, p = 0.00013). The data are mean ± SEM. **p<0.01, N.S., not significant, two-sided Student's t-test (**b-c**). Source data are provided as a Source Data file.

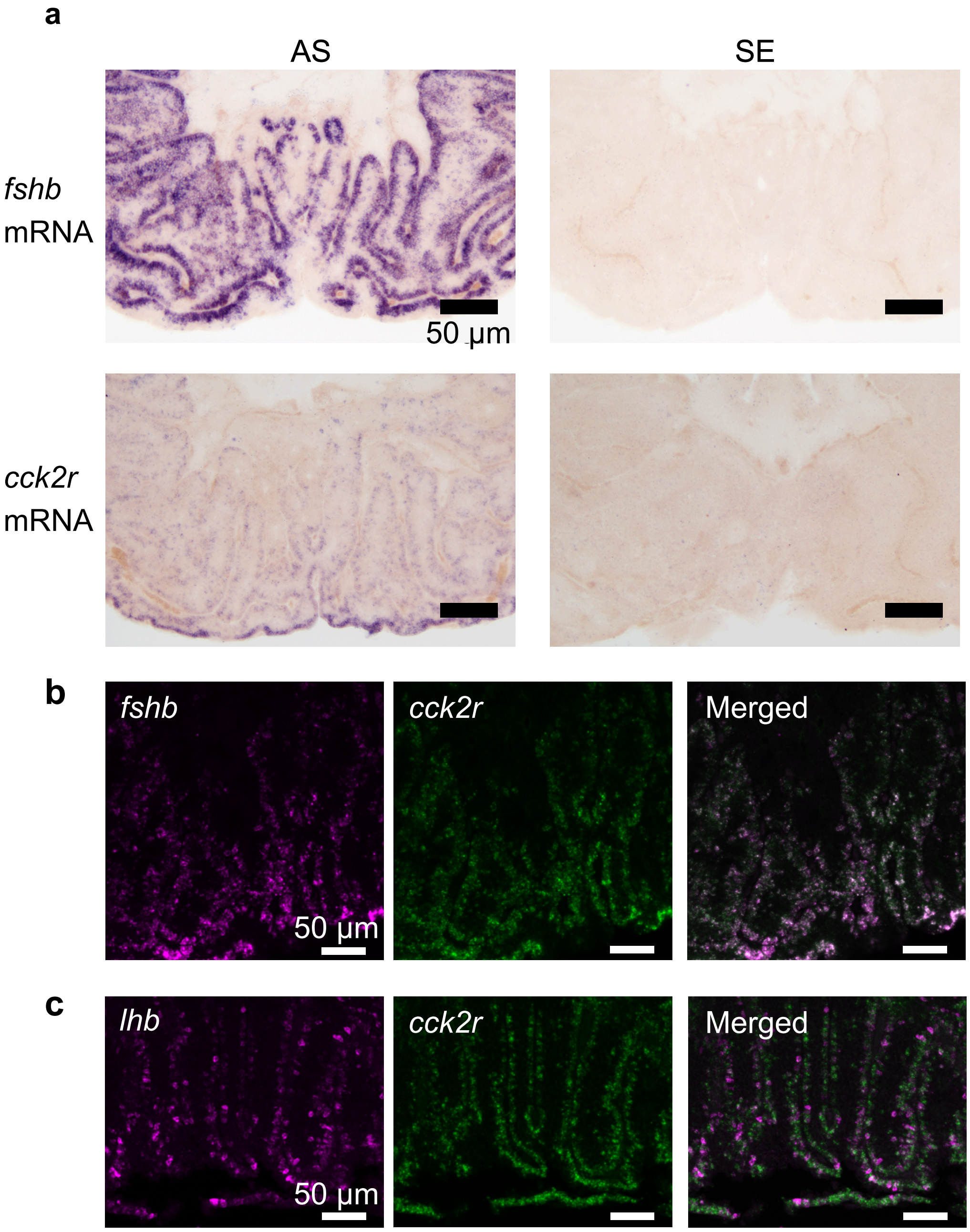

Supplementary Fig. 16. *fshb* and *cck2r* are expressed in the pituitary of Japanese eel.
a *in situ* hybridization of the pituitary in Japanese eel, analyzing the expression of *fshb* and *cck2r* by *in situ* hybridization. Only sections hybridized with anti-sense probe were labeled for both *fshb* and *cck2r*. **b,c** Double *in situ* hybridization of *fshb* (b), *lhb* (c) and *cck2r* (b and c) in the pituitary. Co-expression between *fshb* and *cck2r* was observed, but no co-expression was observed between *lhb* and *cck2r*.

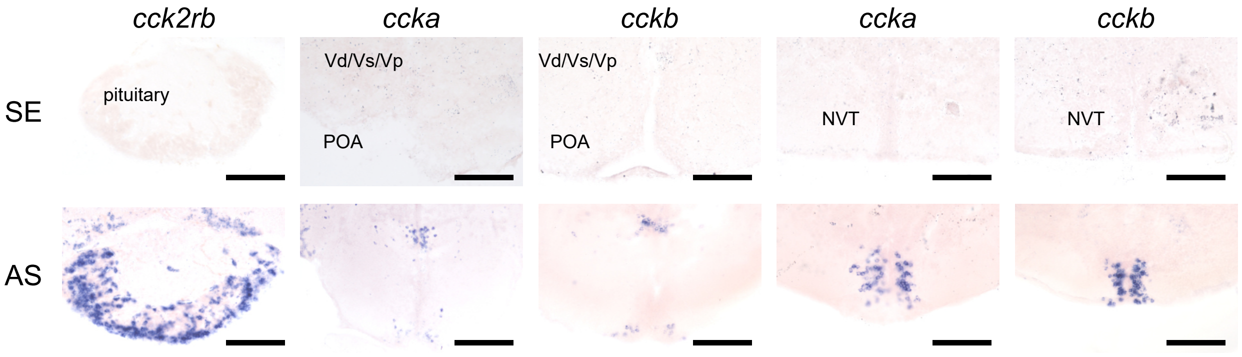

Supplementary Fig. 17. Specific *in situ* hybridization of *cck2rb*, *ccka*, and *cckb*.
in situ hybridization of *cck2rb*, *ccka*, and *cckb*. Only the sections hybridized with anti-sense probes (AS) were labeled whereas sections hybridized with sense probes (SE) did not show any signal which supports the validity of this labeling. Scale bars, 100 μm.

a

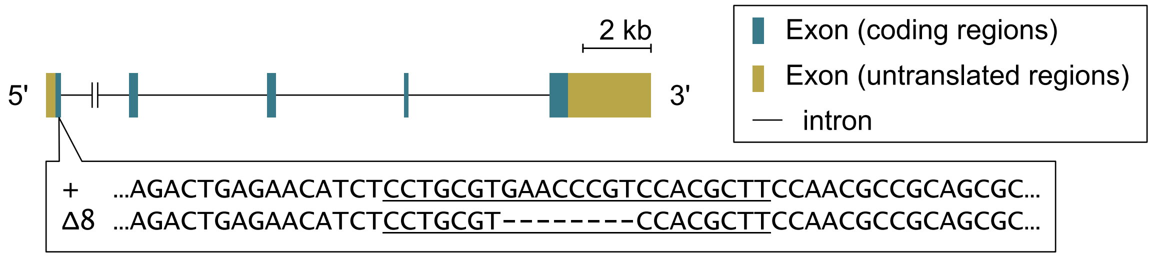

b

```
+   MDTLRNETASTIECVAGALMEQLQSRAAYNGSLETENISCVNPSTLPTPQRVRHREEGHT   60
Δ8  MDTLRNETASTIECVAGALMEQLQSRAAYNGSLETENISCVHASNAAARETQRRGPHAPD   60

+   LRILLYSLIFFLSVFGNLLIIVVLTVNKRMRTVTNTFLLSLAVSDLMMAIFCMPFTLIPN  120
Δ8  PALLAHLLPECLWKPAHHCGPHCQQANAHRH*                               92
+   ILKDFIFGAAMCKIVSYLMGISVSISTFSLVAIAIERYSAICNPLKSRVWQTRSHAYRVI  180

+   AATWVLAFIIMVPYPIISHLEPFQGSANTTAHKCRHKWPIQTAEQTWYVMLLLILFAIPG  240

+   VVMIIAYGLISRELYRGIRFEMGQKKDSTDVKNGLTSTVSNVSDDGDGCYVTVVQRPHSM  300

+   MSTMAASIRQVKADRPRSNTSEAKLEAKKRVIRMLVVIVVLFFLCWMPLYCVNTWRAFDD  360

+   SSATRALSGAPIAFIHLLSYTSACVNPIIYCFMNTRFRKALLSTFSWCGAPCRHCCRRRG  420

+   LRDIEEDVMATGASMSKFSYTTVSTMGNC                                 449
```

Supplementary Fig. 18. Generation of *cck2rb* KO medaka.
a The gene structure of *cck2rb* is shown with the location of the CRISPR target site, which is enlarged to depict the nucleotide sequences of the wild-type (+) and targeted allele (Δ8). The deleted nucleotides are indicated by dashes. **b** Comparison of the deduced Cck2rb protein sequences of the + and targeted allele (Δ8). The transmembrane domain of Cck2rb are shaded in gray. The altered sequences caused by frameshift are indicated in red.

a

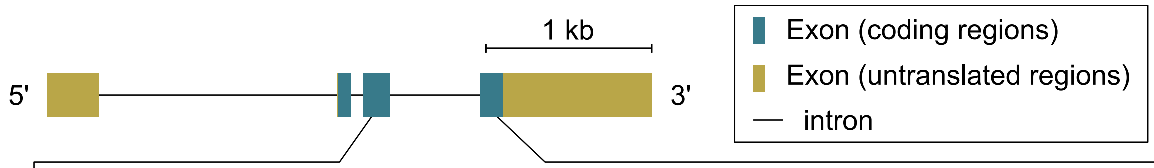


```
+     ...GAAAGCCTACCTGTCCCACTAACCAATCACACGCGTCAGGCTCGCTCTGCTCCTGCGCC
Δ754  ...GAAAGCCTACCTGTCCCACT---------------------------------------

+     CCCTTCAGGACAGCTGCAAAGTGAATCAGCCCAGAACAGCCTGAGCCAGCTGCTGGCCAG
Δ754  ------------------------------------------------------------

+     ACTCCTCTCTAGAAAAAgtgagtgatcagaaacatgtgggcagaaaaccagagaggggaa
Δ754  ------------------------------------------------------------

+     ctgtggcaattgtggacttgatactcatttcgtgttggcatcactgtcccgctcttgttg
Δ754  ------------------------------------------------------------

+     catctcccctcctgacatccgtctgtctgcgggctgccgtcattcacgctgagcccgtac
Δ754  ------------------------------------------------------------

+     cttgccatctggcatttcccacctccctctagcttccgctctccattgtcccgtgccccg
Δ754  ------------------------------------------------------------

+     ctcctgtctcttccataaccgtttcctcaaatcctatccgctgacctgccctttccatta
Δ754  ------------------------------------------------------------

+     ctccctcacacttgtccctacctcctaacctctttctgcctccccatcagcattacaacc
Δ754  ------------------------------------------------------------

+     ctctatcatgactgagtcaacactgtttgacttgcggagctcactgaccttaaagaatat
Δ754  ------------------------------------------------------------

+     aggtccagaaaaacagatcaaatctgcttgagcaactttgacaagttaatttgagaaatc
Δ754  ------------------------------------------------------------

+     agagaataatctcaataaaagtaatactatttagaagaaaataaggctctaacaagctac
Δ754  ------------------------------------------------------------

+     cccccaatgttttctgcttcagGCTCATCCCTCCAGACCAGATCCTCCCTCACCAGCAG
Δ754  ------------------------------------------------------------

+     AGCTGCAGCCCCCAGTCACAGGATAAAGGACAGAGATTACCTGGGATGGATGGACTTTGG
Δ754  -------------------------------------------------------TTTGG

+     GCGACGTAGTGTGGAG...
Δ754  GCGACGTAGTGTGGAG...
```

b

```
+     MNVGIYVCVILAALFTGSLTLPSKSMRTERKALVTESLPVPLTNHTRQARSAPAPPSGQL   60
Δ754  MNVGIYVCVILAALFTGSLTLPSKSMRTERKALVTESLPVPLLGDVVWRSMNIPHKAASF   60

+     QSESAQNSLSQLLARLLSRKSSSLQTRSSLTSRAAAPSHRIKDRDYLGWMDFGRRSVEEY  120
Δ754  ISQHLNQSPKTRFTLLILHQMREDFSSF*                                  89

+     EYSP                                                          124
```

Supplementary Fig. 19. Generation of *ccka* KO medaka.

a The gene structure of *ccka* is shown with the location of the CRISPR target site, which is enlarged to depict the nucleotide sequences of the wild-type (+) and targeted allele (Δ754). The deleted nucleotides are indicated by dashes. **b** Comparison of the deduced Ccka protein sequences of the wild-type (+) and targeted allele (Δ754). The signal peptide region (1-20) and amino acid residue essential for CCK activity are shaded in gray. The altered sequences caused by frameshift are indicated in red.

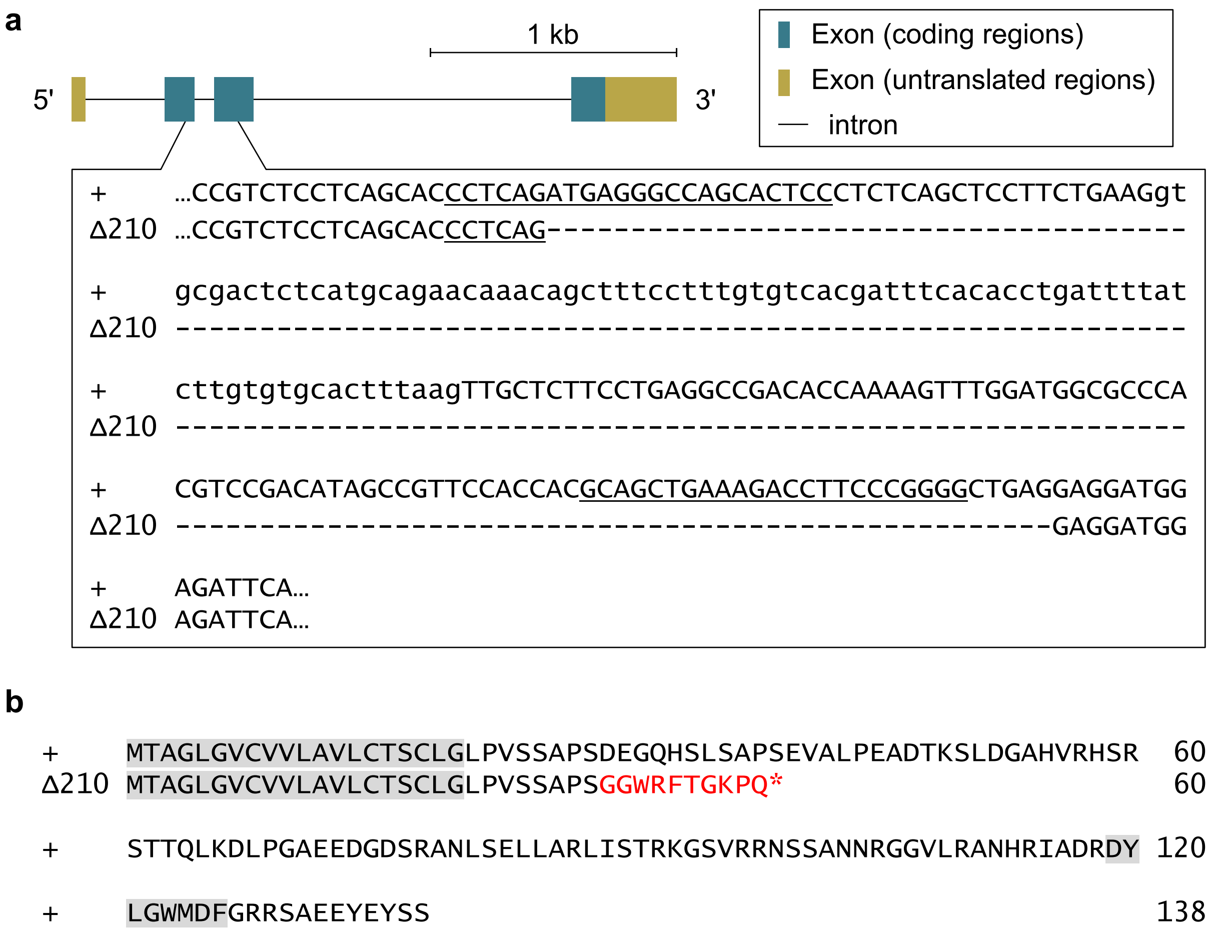

Supplementary Fig. 20. Generation of *cckb* KO medaka.
a The gene structure of *cckb* is shown with the location of the CRISPR target site, which is enlarged to depict the nucleotide sequences of the wild-type (+) and targeted allele (Δ210). The deleted nucleotides are indicated by dashes. **b** Comparison of the deduced Cckb protein sequences of the wild-type (+) and targeted allele (Δ210). The signal peptide region (1-20) and amino acid residue essential for CCK activity are shaded in gray. The altered sequences caused by frameshift are indicated in red.

gene_id	Gene name	annotation	Median TPM value of FSH cell pools (n=4)
ENSORLG00000017966	*cck2rb*	cholecystokinin receptor-like	542.79
ENSORLG00000019757	*gnrh-r2*	gonadotropin-releasing hormone receptor 2	449.315
ENSORLG00000016288	*tgfbr3*	transforming growth factor beta receptor type 3	442.8
ENSORLG00000000865	*chrna4b*	neuronal acetylcholine receptor subunit alpha-2	267.245
ENSORLG00000014011	*pgrmc1*	progesterone receptor membrane component 1	247.1
ENSORLG00000030131	*pk1-r*	pyrokinin-1 receptor-like	224.915
ENSORLG00000024060	*nr1d2b*	nuclear receptor subfamily 1 group D member 2	199.77

Supplementary Table 1. List of highly expressed receptors in the RNAseq analysis of FSH cells

EC50	CCK-8s	Gastrin-8s
pGL4.29(cAMP)	0.2 nM	9.1 nM
pGL4.30(Ca^{2+})	0.7 nM	1.8 nM
pGL4.33(MAPK)	1.0 nM	1.8 nM

Supplementary Table 2. EC50 values of the reporter assay using HeLa cells expressing Cck2rb.

Target	direction	purpose	sequence (5' to 3')
cck2rb	forward	genotyping (gDNA PCR)	AAAGACGGAGAGCCAAAGACAG
cck2rb	reverse	genotyping (gDNA PCR)	TGCAGAGATAGCTTTCTCCAAGAT
cck2rb	forward	genotyping (HRM)	CTGATGGAGCAGCTTCAGAGC
cck2rb	reverse	genotyping (HRM)	TGCTGCGCTTCTCCTGTCGT
cck2rb	forward	genotyping (HRM)	ATCTCCTGCGTGAACCCGTCCACGCTT
ccka	forward	genotyping (gDNA PCR)	GTGTCTAATCCCAATTCAGAAAGTG
ccka	reverse	genotyping (gDNA PCR)	AAGCTGACAAGCTGTGCCTCTT
ccka	forward	genotyping (CS)	ATCCTGGCTGCTCTTTTCACTG
cckb	forward	genotyping (gDNA PCR)	AGCTCCTCACATGACTGAAGCT
cckb	reverse	genotyping (gDNA PCR)	AGTGGCAGGAAAAAGCACTCG
cckb	forward	genotyping (CS)	AAACGCTCCGTCTTCTGTCTGTG
cck2rb	forward	Probe template	CTGATCTCCAGGGAACTTTATCG
cck2rb	reverse	Probe template	GCAGGAGAAAGTATGGAGTACAG
ccka	forward	Probe template	ACTGTTTGAAAGCCTCAGCACCA
ccka	reverse	Probe template	CCCTAGTAGATGATTTGATATGAAGATT
cckb	forward	Probe template	GAACTGCTCTCCTCACTCTCATA
cckb	reverse	Probe template	GCAGCGAAGCAGCTTTTGCTG
ccka	forward	RT-PCR	GCAGTCATGAATGTAGGAATCTACGT
ccka	reverse	RT-PCR	TTATGGGGAATATTCATACTCCTCCACAC
cckb	forward	RT-PCR	TTCTCTCCTCAAGATGACCGCTG
cckb	reverse	RT-PCR	AAGAGTACGAGTACTCCTCATAAGGG
gastrin	forward	RT-PCR	AGGCAGCCATGTCAGGGAAAAC
gastrin	reverse	RT-PCR	TCCCTCCTCCCAAAGTCCA
eel_fshb	forward	Probe template for eel	CAGATTCACAGTTGCCATGCATCT
eel_fshb	reverse	Probe template for eel	CATCTATCCCTTGCCGCAGTT
eel_cck2r	forward	Probe template for eel	GCGATGGACGTACAGAAACTGAATG
eel_cck2r	reverse	Probe template for eel	CTTCCAGGTGTTGACGGAGTAG
cck2rb	forward	cds cloning (including sequence for construction)	CTTGCCGCCATGGATACTTTGAGAAACG AGAC
cck2rb	reverse	cds cloning (including sequence for construction)	CTCTCAGCAGTTTCCCATGGTG
actb	forward	qPCR	GTGATGTTGATATCCGTAAGGATCTGTA
actb	reverse	qPCR	TCTGGTGGGGCAATGATCTTGA
fshb	forward	qPCR	TGGAGATCTACAGGCGTCGGTAC

fshb	reverse	qPCR	AGCTCTCCACAGGGATGCTG
lhb	forward	qPCR	TGCCTTACCAAGGACCCCTTGATG
lhb	reverse	qPCR	AGGGTATGTGACTGACGGATCCAC
tshb	forward	qPCR	GCTACTCAAGGGACAGCA
tshb	reverse	qPCR	GCAGCCTCTCTGGATAAGGAA
*fsh*UP+	Forward	Construct for rescue	CAGAGGGGAGCCACCATGGATACTTTGA
*cck2rb*SE		transgenic	GAAACGAGACAGC
*Cck2rb*FLA	Reverse	Construct for rescue	GTCGCGGCCGCTTCATCACTTATCGTCGT
Gstop+		transgenic	CATCCTTGTAATCTCCTCCGCAGTTTCCC
LinkAS			ATGGTGCTG

Supplementary Table 3. Primers used in this study.

gDNA PCR, PCR on genomic DNA; CS, cycle sequence; HRM, high-resolution melt analysis. Unless otherwise specified, all target genes mentioned are from the medaka.
