# Supplementary material for: Identification of the FSH-RH as the other gonadotropin-releasing hormone
Source: Nat Commun. 2024 Jun 27;15:5342. doi: 10.1038/s41467-024-49564-8 (PMC11211334; doi:10.1038/s41467-024-49564-8)
Supplement: Supplementary file 3 — Reporting Summary [file 41467_2024_49564_MOESM3_ESM.pdf]

nature portfolio

Corresponding author(s): Kanda Shinji

Last updated by author(s): May 20, 2024

Reporting Summary

Nature Portfolio wishes to improve the reproducibility of the work that we publish. This form provides structure for consistency and transparency in reporting. For further information on Nature Portfolio policies, see our Editorial Policies and the Editorial Policy Checklist.

Statistics

For all statistical analyses, confirm that the following items are present in the figure legend, table legend, main text, or Methods section.

n/a	Confirmed	
☐	☒	The exact sample size (*n*) for each experimental group/condition, given as a discrete number and unit of measurement
☐	☒	A statement on whether measurements were taken from distinct samples or whether the same sample was measured repeatedly
☐	☒	The statistical test(s) used AND whether they are one- or two-sided<br>*Only common tests should be described solely by name; describe more complex techniques in the Methods section.*
☒	☐	A description of all covariates tested
☐	☒	A description of any assumptions or corrections, such as tests of normality and adjustment for multiple comparisons
☐	☒	A full description of the statistical parameters including central tendency (e.g. means) or other basic estimates (e.g. regression coefficient) AND variation (e.g. standard deviation) or associated estimates of uncertainty (e.g. confidence intervals)
☐	☒	For null hypothesis testing, the test statistic (e.g. *F*, *t*, *r*) with confidence intervals, effect sizes, degrees of freedom and *P* value noted<br>*Give P values as exact values whenever suitable.*
☒	☐	For Bayesian analysis, information on the choice of priors and Markov chain Monte Carlo settings
☒	☐	For hierarchical and complex designs, identification of the appropriate level for tests and full reporting of outcomes
☒	☐	Estimates of effect sizes (e.g. Cohen's *d*, Pearson's *r*), indicating how they were calculated

Our web collection on statistics for biologists contains articles on many of the points above.

Software and code

Policy information about availability of computer code

Data collection	Micromanager, LightCycler480 Software, Olympus FV10-ASW, Leica Application Suite X
Data analysis	ImageJ, R, Kyplot 6.0, Microsoft Excel, MEGA 11, GeneDoc 2.7.000

For manuscripts utilizing custom algorithms or software that are central to the research but not yet described in published literature, software must be made available to editors and reviewers. We strongly encourage code deposition in a community repository (e.g. GitHub). See the Nature Portfolio guidelines for submitting code & software for further information.

Data

Policy information about availability of data

All manuscripts must include a data availability statement. This statement should provide the following information, where applicable:

- Accession codes, unique identifiers, or web links for publicly available datasets
- A description of any restrictions on data availability
- For clinical datasets or third party data, please ensure that the statement adheres to our policy

The authors confirm that all relevant data are provided in this paper, in its Supolementary Information files, or public data stroage shown in the text.

Research involving human participants, their data, or biological material

Policy information about studies with human participants or human data. See also policy information about sex, gender (identity/presentation), and sexual orientation and race, ethnicity and racism.

Reporting on sex and gender	N/A
Reporting on race, ethnicity, or other socially relevant groupings	N/A
Population characteristics	N/A
Recruitment	N/A
Ethics oversight	N/A

Note that full information on the approval of the study protocol must also be provided in the manuscript.

Field-specific reporting

Please select the one below that is the best fit for your research. If you are not sure, read the appropriate sections before making your selection.

☒ Life sciences ☐ Behavioural & social sciences ☐ Ecological, evolutionary & environmental sciences

For a reference copy of the document with all sections, see nature.com/documents/nr-reporting-summary-flat.pdf

Life sciences study design

All studies must disclose on these points even when the disclosure is negative.

Sample size	In all experiments using medaka, sample size calculations were carried out to obtain 0.05 significance.
Data exclusions	One of the fshb expresssion of cck2rb+/- male data was excluded because of non specific amplification in real time PCR indicated from melting curve. No other data is excluded.
Replication	All experiments shown are the primary experiments conducted after the preliminary test.
Randomization	Medaka were randomly assigned to experimental conditions.
Blinding	Experimenters were not blind to group allocation during data acquisition or analysis except for Ca2+ imaging. Controls and experiments group were tested in the same condition, and data were analyzed with the same criteria.

Reporting for specific materials, systems and methods

We require information from authors about some types of materials, experimental systems and methods used in many studies. Here, indicate whether each material, system or method listed is relevant to your study. If you are not sure if a list item applies to your research, read the appropriate section before selecting a response.

Materials & experimental systems

n/a	Involved in the study	
☐	☒	Antibodies
☐	☒	Eukaryotic cell lines
☒	☐	Palaeontology and archaeology
☐	☒	Animals and other organisms
☒	☐	Clinical data
☒	☐	Dual use research of concern
☒	☐	Plants

Methods

n/a	Involved in the study	
☒	☐	ChIP-seq
☒	☐	Flow cytometry
☒	☐	MRI-based neuroimaging

Antibodies

Antibodies used	Anti-DIG-POD, anti-DIG-AP, anti-fluorescein, Anti-Cholecystokinin (CCK8) antibody (C2581,Sigma), DDDDK-tag antibody (PM020, MLB)

Validation	Immunohistochemistry of ccka and cckb double KO validated the specificity of C2581.DDDDK-tag antibody was confirmed to label no cell in the WT sample.<br>Sense and antisense probes validated the specificity of antibodies used in ISH.

Eukaryotic cell lines

Policy information about cell lines and Sex and Gender in Research

Cell line source(s)	HeLa cells from Riken BRC Cell Bank (Cell number: RCB0007) were used in this study. HeLa cells were established from the cervix cells of a female human.
Authentication	HeLa cells were authenticated by short tandem repeat (STR) profiling (National Institute of Biomedical Innovation, Osaka, Japan)
Mycoplasma contamination	HeLa cells were confirmed to be mycoplasma free (Biotherapy Institute of Japan, Tokyo, Japan).
Commonly misidentified lines<br>(See ICLAC register)	No commonly misidentified cell lines were used in the study.

Animals and other research organisms

Policy information about studies involving animals; ARRIVE guidelines recommended for reporting animal research, and Sex and Gender in Research

Laboratory animals	d-rR strain medaka (Oryzias latipes), himedaka (Oryzias latipes) and Japanese eel (Anguilla japonica)
Wild animals	No wild animals are used.
Reporting on sex	Sex was determined by the color and fin shapes of the sample
Field-collected samples	No field-collected samples are used
Ethics oversight	Experiments were performed at the Atmosphere and Ocean Research Institute and Department of Aquatic Bioscience of Graduate school of agricultural and life sciences of the university of the Tokyo in accordance with the protocols approved by the animal care and use committee of Atmosphere and Ocean Research Institute of the University of Tokyo (Permission number, P22-6, P22-7).

Note that full information on the approval of the study protocol must also be provided in the manuscript.

Plants

Seed stocks	N/A
Novel plant genotypes	N/A
Authentication	N/A
